# RegNetB: Predicting Relevant Regulator-Gene Relationships in Localized Prostate Tumor Samples

**DOI:** 10.1186/1471-2105-12-243

**Published:** 2011-06-17

**Authors:** Angel Alvarez, Peter J Woolf

**Affiliations:** 1 Department of Chemical Engineering, University of Michigan, Ann Arbor, MI 48109, USA; 2 Department of Biomedical Engineering, University of Michigan, Ann Arbor, MI 48109, USA; 3 Bioinformatics Program, University of Michigan, Ann Arbor, MI 48109, USA

## Abstract

**Background:**

A central question in cancer biology is what changes cause a healthy cell to form a tumor. Gene expression data could provide insight into this question, but it is difficult to distinguish between a gene that causes a change in gene expression from a gene that is affected by this change. Furthermore, the proteins that regulate gene expression are often themselves not regulated at the transcriptional level. Here we propose a Bayesian modeling framework we term RegNetB that uses mechanistic information about the gene regulatory network to distinguish between factors that cause a change in expression and genes that are affected by the change. We test this framework using human gene expression data describing localized prostate cancer progression.

**Results:**

The top regulatory relationships identified by RegNetB include the regulation of RLN1, RLN2, by PAX4, the regulation of ACPP (PAP) by JUN, BACH1 and BACH2, and the co-regulation of PGC and GDF15 by MAZ and TAF8. These target genes are known to participate in tumor progression, but the suggested regulatory roles of PAX4, BACH1, BACH2, MAZ and TAF8 in the process is new.

**Conclusion:**

Integrating gene expression data and regulatory topologies can aid in identifying potentially causal mechanisms for observed changes in gene expression.

## Background

What changes are responsible for making a tumor a tumor? If we knew the underlying cause for this change, then it may be possible to directly address the underlying dysfunction that causes tumorigenesis. One possible route to identifying a causal mechanism for tumorigenesis is to gather a rich body of experimental data describing the state of many tumors and search for relevant signatures. Unfortunately, it is difficult to distinguish the signatures that are a consequence of the dysfunction from the signatures that cause the dysfunction.

A further complication is that the activity of the factors that influence gene expression is difficult to observe directly. For example, consider the simplest case of a single transcription factor that regulates the expression of one target gene. In this case, the activity of the transcription factor may be governed by its past history of mRNA expression, possible splice variants, protein modification, binding with other factors, and where the transcription factor is localized in the cell. In this case, the most direct measure of the activity of the transcription factor is the expression of the target gene itself. However, when multiple genes are coordinately regulated by multiple regulators, analyzing these cause and effect relationships becomes more difficult.

One source of information relating transcription factors to their target genes is the transcription factor-DNA binding information in databases such as TRANSFAC and MsigDB [1,2]. However, knowing transcription factor-DNA binding relationships alone does not identify which regulatory activities are relevant for a specific disease or tissue under study [3,4]. This limitation can be partially overcome if gene expression data are integrated with transcription factor-DNA binding information to identify which transcriptional activities better explain the observed expression variation.

### Regulatory Networks-Bayesian (RegNetB)

In this work, we have developed and tested a tool called Regulatory Networks-Bayesian, or RegNetB, to carry out this integration of gene expression data and transcription factor-DNA binding information. RegNetB uses a simplified topology to describe a regulatory network in which the top layer of this network represents the group of unobserved regulators (transcription factor activities) and the bottom layer represents observed genes (mRNA expression values). This regulatory bipartite network model has been used elsewhere to represent transcriptional regulatory networks by adopting a linear mixing model [5-8]. Other methods such as NIR, CRL and ARACNE also attempt to identify transcription factors-genes associations [9-11], however these methods make a number of simplifying assumptions that limit their applicability. In all cases, the activities of the transcription factors is assumed to be proportional to the expression level of the transcription factor--an assumption that ignores the possible post translational regulation of transcription factor activity. Furthermore, in the case of NIR, linear modeling is used for this identification even though transcriptional regulatory relationships could be more complex. CRL and ARACNE adopt a mutual information based approach to account for non-linearities. However, the mutual information approach used in CRL and ARACNE is limited to pairwise interactions and as such would miss higher order phenomena. TF-Finder is another method recently proposed that uses correlation and linear combination maximization to propose a list of potential transcription factors associated with the biological processes described by the expression data used [12]. Here we extend these models to account for both linear and nonlinear influences from a variable number of unobserved regulators using a full Bayesian approach [13-15].

A common criticism when using Bayesian networks on gene expression data is that loops are not permitted in a Bayesian network structure. Nevertheless, because we are not adopting a model where the activity of a transcription factors is equivalent to its gene expression data, we can represent the activity of transcription factor "A" (in the top layer) regulating the expression of gene "A" (bottom layer) without posing a violation on the Bayesian networks loops limitation. In other words, in our model, the activity of transcription factor "A" and the expression of gene "A" represent two different variables. This same rationale applies to other structures like FFL (Feed Forward Loops)[16] that might be challenging to work with a traditional Bayesian networks analysis.

RegNetB is tested using gene expression data from a prostate cancer study carried out elsewhere [17,18]. Despite the high incidence and mortality rate, the molecular mechanisms underlying the oncogenesis and progression of prostate cancer are still unclear. Significant research has been dedicated to identifying prognostic markers, however less research has focused on identifying the regulatory mechanism that drives the disease [19].

By identifying a group of the most relevant regulatory relationships, RegNetB is able to identify which regulators are most likely responsible for the expression variations in the prostate cancer study evaluated here. In the next sections we describe the data processing and results obtained after RegNetB analysis.

## Methods

In the following section, we will describe the RegNetB algorithm and the data preprocessing used in our test cases.

### RegNetB algorithm

The transcription factor-gene network presented here is modeled as a Bayesian network by RegNetB. Regulators in this network are modeled as hidden variables and the observed variables (genes) are modeled using a multinomial model with Dirichlet priors as described elsewhere [13-15] and in the supplemental material Additional file 1: BDE_scoring_metric.doc. Below we provide a summary of the scoring process.

For a typical Bayesian network scoring problem, a complete discrete data set describing the variables included in the network of interest is available. However, in this case the transcription factors are not observed. To fill in the activity levels for the regulators, a Gibbs sampler is used to sample over the space unobserved regulators [20-23]. Gibb's sampling and network scoring were carried out using PEBL, a python library developed in our research group [24]. PEBL estimates the probability of a discretized dataset given a specific network using a Bayesian Dirichlet equivalent metric described elsewhere [13]. The source code of PEBL can be freely downloaded from (http://code.google.com/p/pebl-project/).

Two scoring steps are performed by RegNetB to evaluate the relative strength of each connection in the transcription factor-gene network. First, sample states of the unobserved transcription factors are taken using a Gibbs sampler. The sample states are taken after a burn in of 10 iterations. The second scoring step uses these sample states to rescore the whole network when each transcription factor-gene edge is removed and then re-added. The relative importance of the edge can then be interpreted as the change in the average score of the network when the edge is removed versus present. Source code for this scoring procedure is provided in the additional file 2: RNB_scoring.py.

To generate the final list of regulators and genes of interest in our study, we first ranked all the connections based on the scores estimated by RegNetB. After normalizing all the connection scores, a graphical analysis was used to identify thresholds that differentiate a group of relatively stronger connections from the rest based on their scores. A list of all the genes and regulators was generated from this set of connections.

### Global human transcription factor-gene network

A global human transcription factor-gene network was created using the Molecular Signatures Database (MsigDB) [2]. The source of the "C3: Motif Gene Set" information in this database, the collection we used to create the global human transcription network, is described elsewhere [25]. Briefly, the transcription factor binding sites were predicted using promoter sequence analysis, gene set enrichment analysis (GSEA), and comparative genomic analysis. After collecting these transcription factor binding sites and the genes associated with them, the gene names were mapped to their official Entrez gene symbols. Only those genes mapping to unique official gene symbols were included. Similarly, some binding sites mapped to known transcription factors (regulators) names documented in TRANSFAC while others were only described as the sequence of the promoter itself. Regulatory sequences not mapping to any known regulator were listed as UK (unknown) followed by an integer.

### Gene expression data

We used RegNetB to analyze 146 gene expression profiles from prostate tissue samples described elsewhere [17,18] and available online on GEO as GDS2545. This set of expression profiles includes 18 profiles from normal prostate tissues, 63 profiles from normal prostate tissues adjacent to localized tumor, and 65 profiles from primary prostate cancer tumors. The 146 gene expression profiles were pre-processed using the web-based genechip analysis system (WGAS) described elsewhere [26,27] for data normalization and mapping of probe sets ID to official gene symbols.

Next we filtered the gene list to only include genes that could be meaningfully analyzed. The genes passing the filter must: (1) exhibit differential expression across the samples; (2) be present in the global human transcription network; and (3) not have more than 10 regulators as parents in the global human transcription network. The first criterion was satisfied by selecting the top 500 genes with the largest variation as measured by the magnitude of the standard deviation of the expression values across samples. The second and third criteria were then applied to this list of 500 genes to identify genes in the network with 10 or fewer regulators. We note that while it is possible that a gene with more than 10 regulators could mechanistically participate in a strong regulatory relationship, this relationship will not be identifiable with a small dataset in a multinomial model such as we are using here. In a multinomial model, the number of parameters increases exponentially with the number of regulators, making any relationship in a highly connected gene weak. As such, by eliminating genes with more than 10 regulators we are eliminating genes that are unlikely to score well.

### Data discretization

The scoring metric used by RegNetB requires that the data be discretized. The data for this study were binned into three states describing a high, medium and low expression level for the variables. The bin sizes were evenly distributed across samples for each variable generating a discretized data set in which variables have their top 1/3 of the data entries based on expression as "high", the bottom 1/3 of the entries as "low" and the remainder 1/3 of the entries as "medium". This binning strategy (3 bins and evenly distributed) has been used elsewhere and has been shown empirically to be robust in capturing relevant details of the systems under study [11,28-31]. In addition to following these strategies that are becoming a standard in the systems biology community, there is a strong computational incentive to keep the number of discretization bins as small as possible. This incentive arises because RegNetB uses a Gibbs sampling approach to explore the possible configurations of the unobserved regulators. As the number of bins increases, the size of the search space increases exponentially. For example, for a dataset with 100 observations and two unobserved regulators, the configuration space of the unobserved regulators is 3^100*2 ^~ 10^95 ^for a 3 bin discretization, and 4^100*2 ^~ 10^125 ^for a 4 bin discretization--an increase by a factor of 10^30^!

## Results and Discussion

### Global human transcription factor-gene network

The final global human transcription factor-gene bipartite network generated from the MsigDB consists of 12,015 gene symbols and 391 regulators with a total of 134,573 regulator-gene associations. From these 391 regulators or regulatory regions, 216 were associated with known transcription factors names. The remaining 175 regulators (UK1 to UK175) consisted of 60 known regulatory sequences documented in TRANSFAC and 115 regulatory sequences found and documented elsewhere [25]. This global bipartite human transcription network is included in the supplemental material additional file 3: global_human_transcription_net.xlsx. After filtering, we compiled a final list of 253 genes and 292 regulators interconnected in a bipartite network with 1,266 connections.

### Strongest connections identified by RegNetB

**Figure 1:**
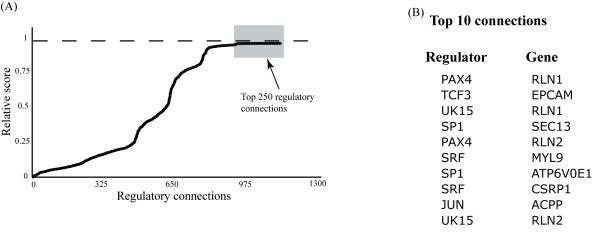
**Connections ranked by score**. (A) Relative score distribution for the regulatory connections kept after RegNetB analysis. The shadowed region shows the top 250 connections based on score.. (B) Top 10 connections predicted by RegNetB.

Figure 1(A) shows the score distribution of transcription factor-gene connections. Based on this distribution, we selected the connections that ranked at the top area of the curve illustrated in the Figure 1(A). This group of regulatory connections shows a clear similarity in terms of the regulatory strength. A total of approximately 250 regulatory connections were collected, all with a score >0.993, and are listed in the supplemental material additional file 4: top_regulatory_assoc.xls. Figure 1(B) shows the top 10 connections from this list.

We evaluated the probability that a regulatory association was picked within the top best 10, 50, 100 and 250 based on score when random values were assigned to the regulator's activities instead of the suggested sampling from the expression data used by RegNetB (no signal). This random-value test was run 5000 times. Estimation of p-values < 0.05 supported that the selection of all the top 10, 50 and 100 edges by RegNetB was not by a random event. For the best top 250 edges, 70% of the edges showed p-values < 0.05 supporting their selection from a non-random event. In addition, after three independent runs of RegNetB on the same dataset/initial network, more than 95% of the top 250 associations based on score were consistently predicted in the three runs, with most of the 5% inconsistencies ranking in positions 225-275 (borderline).

After analysis of the posterior distribution of the networks scores, the top 218 networks based on score showed statistically to be different (better) than the rest. For this analysis, we compared each "edge" disconnection score to the initial network score. This score comparison tested which connections, compared to all others, showed to negatively affect more the score of the initial network when they were disconnected (more relevant edges). The top 218 edges based on score showed a p-value < 0.05. This is consistent with the results explained in the previous paragraph.

We also noticed that not all connections associated with a regulator included in the top 250 strongest connections list were part of the group of top connections. This relative strength distribution implies that some regulatory connections associated with a specific regulator play a more relevant regulatory function than the others.

### PAX4 regulatory role

Regulation of RLN1 by PAX4 ranked top on the list of strong connections in Figure 1(B). Similarly, the regulation of RLN2 by PAX4 also ranked well (fifth position). RLN1 and RLN2 have been associated with prostate cancer in other studies [32]. The regulator PAX4 has been identified as a tumor suppressor in melanoma studies [33], however has not been associated with prostate cancer [34].

**Figure 2:**
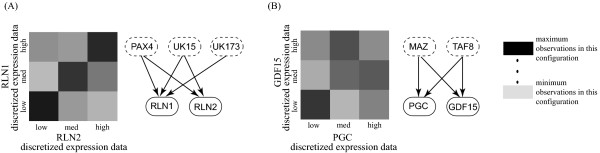
**Top scoring regulatory relationships and discretized data patterns**. Each grid in (A) and (B) shows the nine possible state combinations in which each pair of variables is observed in the discretized expression data. In the regulatory networks, the dotted ovals represent regulators while the solid ovals represent target genes. (A) RLN1 and RLN2 expression and regulatory network. Note that RLN1 and RLN2 show a nearly linear co-expression pattern. (B) PGC and GDF15 expression and regulatory network. The expression pattern of PGC relative to GDF15 does not show a linear pattern, but still scores well in the multinomial model used by RegNetB.

To further evaluate the RegNetB prediction of PAX4's influence on RLN1 and RLN2, we examined the expression levels of the target genes and any other regulator(s) associated with the genes. As shown in Figure 2(A), the expression patterns of RLN1 and RLN2 share a strong similarity in terms of regulation not only by the topological model but also by the coordinated linear pattern observed in the data. This observation supports the prediction that PAX4 is a common factor responsible for changes in the expression of RLN1 and RLN2.

### ACPP (PAP) regulation

Another connection observed in the strong connection list shown in Figure 1(B) was the regulation of ACPP by JUN. ACPP or PAP (Prostatic Acid Phosphatase) is a known prostate cancer marker used to monitor tumor progression and/or patients improvement [35]. RegNetB suggested that the main regulatory activity associated with this gene is best described by the regulators JUN, BACH1, and BACH2. JUN is an oncogene that has been associated with different types of cancer including prostate cancer tumor progression [36,37]. In the case of BACH1 and BACH2, even though there are some associations with breast cancer and leukemia[38,39], we found no links with prostate tumor progression.

### MAZ and TAF8 co-regulation

To further explore RegNetB's results, we examined sets of two or more genes that shared the same group of regulators within the selected list of 250 regulatory connections. We found two genes, PGC and GDF15 that are both co-regulated by TAF8 and MAZ. Both PGC and GDF15 have been associated with prostate cancer and have been documented as potential biomarkers [40-42]. Figure 2(B) shows coordinated patterns between these genes but not in a linear manner. Interestingly, MAZ and TAF8 have been associated with other types of cancer [43-45], but we found no reports associating MAZ and TAF8 with prostate cancer.

## Conclusions

These results suggest that RegNetB is able to identify physiologically relevant regulatory protein-gene relationships based on gene expression data. Many of the target genes identified by RegNetB have been implicated in prostate cancer progression, but the relevant regulation is largely new. In particular, RegNetB identified the regulators PAX4, BACH1, BACH2, MAZ and TAF8 as playing a central role in this prostate cancer gene expression data set. Most of the significant associations predicted in this work are currently being experimentally evaluated.

The method used by RegNetB can be directly applied to any gene expression dataset, as long as a transcriptional regulatory network is known for the organism. We acknowledge that gene regulations are hardly a one-step process and other genes beside the ones predicted by RegNetB will change as well because they were not part of the initial network or because of others regulatory events not captured by the integrated data used. What we intended in this work was to present a systematic procedure to filter and predict a set of regulatory associations that more likely explain most of the changes in expression. By identifying such explanatory regulatory protien-gene relationships, RegNetB allows a researcher to look beyond changes in gene expression, and start to identify possible causes for that change in expression.

## Competing interests

The authors declare that they have no competing interests.

## List of Abbreviations

RegNetB: Regulatory Networks-Bayesian.

## Authors' contributions

AA Designed the RegNetB approach and software, carried out the analysis, and wrote the paper

PJW Designed the RegNetB approach, guided the analysis, and edited the paper

All authors read and approved the final manuscript.

## Supplementary Material

Additional file 1 **Bayesian Dirichlet metric**. This document includes more details about the BD scoring metric used in this work as well as an explanation of the Gibbs sampler integrated for the scoring in the presence of hidden variables or missing entries in general. Click here for file

Additional file 2 **RegNetB scoring script**. This file contain the script used to sample the hidden variables and score all the networks resulting from each edges disconnections to estimate each edge relative strength. Click here for file

Additional file 3 **Global human bipartite transcription network**. This file contains all the regulatory associations gathered from the MsigDB database and a map for the regulators identified as "UK" (unknown) with their respective promoter regulatory sequence. Click here for file

Additional file 4 **Top 250 regulatory associations found in this work**. This file contains a table with the list of the top-ranked regulatory associations based on score found by RegNetB. Click here for file

## References

[B1] Matys V Kel-Margoulis OV Fricke E Liebich I Land S Barre-Dirrie A Reuter I Chekmenev D Krull M Hornischer K TRANSFAC and its module TRANSCompel: transcriptional gene regulation in eukaryotes Nucleic Acids Res 2006 34 Database issue D108 110 16381825 10.1093/nar/gkj143 PMC1347505

[B2] Subramanian A Tamayo P Mootha VK Mukherjee S Ebert BL Gillette MA Paulovich A Pomeroy SL Golub TR Lander ES Gene set enrichment analysis: a knowledge-based approach for interpreting genome-wide expression profiles Proc Natl Acad Sci USA 2005 102 43 15545 15550 10.1073/pnas.0506580102 16199517 PMC1239896

[B3] Bulyk ML Computational prediction of transcription-factor binding site locations Genome Biol 2003 5 1 201 10.1186/gb-2003-5-1-201 14709165 PMC395725

[B4] Kel AE Gossling E Reuter I Cheremushkin E Kel-Margoulis OV Wingender E MATCH: A tool for searching transcription factor binding sites in DNA sequences Nucleic Acids Res 2003 31 13 3576 3579 10.1093/nar/gkg585 12824369 PMC169193

[B5] Brynildsen MP Wu TY Jang SS Liao JC Biological network mapping and source signal deduction Bioinformatics 2007 23 14 1783 1791 10.1093/bioinformatics/btm246 17495996

[B6] Beal MJ Ghahramani Z Variational Bayesian Learning of Directed Graphical Models with Hidden Variables Bayesian Analysis 2006 1 4 40

[B7] Sabatti C James GM Bayesian sparse hidden components analysis for transcription regulation networks Bioinformatics 2006 22 6 739 746 10.1093/bioinformatics/btk017 16368767

[B8] Pe'er D Regev A Tanay A Minreg: A Scalable Algorithm for Learning Parsimonious Regulatory networks in Yeast and Mammals Journal of Machine Learning Research 2006 7 167 189

[B9] Gardner TS di Bernardo D Lorenz D Collins JJ Inferring genetic networks and identifying compound mode of action via expression profiling Science 2003 301 5629 102 105 10.1126/science.1081900 12843395

[B10] Faith JJ Hayete B Thaden JT Mogno I Wierzbowski J Cottarel G Kasif S Collins JJ Gardner TS Large-scale mapping and validation of Escherichia coli transcriptional regulation from a compendium of expression profiles PLoS Biol 2007 5 1 e8 10.1371/journal.pbio.0050008 17214507 PMC1764438

[B11] Margolin AA Nemenman I Basso K Wiggins C Stolovitzky G Dalla Favera R Califano A ARACNE: an algorithm for the reconstruction of gene regulatory networks in a mammalian cellular context BMC Bioinformatics 2006 7 Suppl 1 S7 10.1186/1471-2105-7-S1-S7 16723010 PMC1810318

[B12] Cui X Wang T Chen HS Busov V Wei H TF-finder: a software package for identifying transcription factors involved in biological processes using microarray data and existing knowledge base BMC Bioinformatics 2010 11 425 10.1186/1471-2105-11-425 20704747 PMC2930629

[B13] Cooper GF Herskovits E A Bayesian method for the induction of probabilistic networks from data Machine Learning 1992 9 4 39

[B14] Sachs K Gifford D Jaakkola T Sorger P Lauffenburger DA Bayesian network approach to cell signaling pathway modeling Sci STKE 2002 2002 148 pe38 12209052 10.1126/stke.2002.148.pe38

[B15] Woolf PJ Prudhomme W Daheron L Daley GQ Lauffenburger DA Bayesian analysis of signaling networks governing embryonic stem cell fate decisions Bioinformatics 2005 21 6 741 753 10.1093/bioinformatics/bti056 15479714

[B16] Milo R Shen-Orr S Itzkovitz S Kashtan N Chklovskii D Alon U Network motifs: simple building blocks of complex networks Science 2002 298 5594 824 827 10.1126/science.298.5594.824 12399590

[B17] Chandran UR Ma C Dhir R Bisceglia M Lyons-Weiler M Liang W Michalopoulos G Becich M Monzon FA Gene expression profiles of prostate cancer reveal involvement of multiple molecular pathways in the metastatic process BMC Cancer 2007 7 64 10.1186/1471-2407-7-64 17430594 PMC1865555

[B18] Yu YP Landsittel D Jing L Nelson J Ren B Liu L McDonald C Thomas R Dhir R Finkelstein S Gene expression alterations in prostate cancer predicting tumor aggression and preceding development of malignancy J Clin Oncol 2004 22 14 2790 2799 10.1200/JCO.2004.05.158 15254046

[B19] Abate-Shen C Shen MM Molecular genetics of prostate cancer Genes Dev 2000 14 19 2410 2434 10.1101/gad.819500 11018010

[B20] Heckerman D Learning in Graphical Models 1999 MIT Press, Cambridge, MA

[B21] Ghahramani Z An introduction to hidden Markov models and Bayesian networks Hidden Markov models: applications in computer vision 2002 World Scientific Publishing Co., Inc 9 42

[B22] Gilks WR Markov Chain Monte Carlo in Practice 1995

[B23] Riggelsen C Learning parameters of Bayesian networks from incomplete data via importance sampling International Journal of Approximate Reasoning 2006 42 1-2 15

[B24] Shah A Woolf PJ Python Environment for Bayesian Learning: Inferring the Structure of Bayesian Networks from Knowledge and Data Journal of Machine Learning Research 2009 10 4 PMC2804996 20161541

[B25] Xie X Lu J Kulbokas EJ Golub TR Mootha V Lindblad-Toh K Lander ES Kellis M Systematic discovery of regulatory motifs in human promoters and 3' UTRs by comparison of several mammals Nature 2005 434 7031 338 345 10.1038/nature03441 15735639 PMC2923337

[B26] Dai M Wang P Jakupovic E Watson SJ Meng F Web-based GeneChip analysis system for large-scale collaborative projects Bioinformatics 2007 23 16 2185 2187 10.1093/bioinformatics/btm297 17586830

[B27] Dai M Wang P Boyd AD Kostov G Athey B Jones EG Bunney WE Myers RM Speed TP Akil H Evolving gene/transcript definitions significantly alter the interpretation of GeneChip data Nucleic Acids Res 2005 33 20 e175 10.1093/nar/gni179 16284200 PMC1283542

[B28] Friedman N Probabilistic models for identifying regulation networks Bioinformatics 2003 19 suppl_2 ii57

[B29] Yu J Smith VA Wang PP Hartemink AJ Jarvis ED Advances to Bayesian network inference for generating causal networks from observational biological data Bioinformatics 2004 20 18 3594 3603 10.1093/bioinformatics/bth448 15284094

[B30] Friedman N Inferring cellular networks using probabilistic graphical models Science 2004 303 5659 799 805 10.1126/science.1094068 14764868

[B31] Friedman N Linial M Nachman I Pe'er D Using Bayesian networks to analyze expression data J Comput Biol 2000 7 3-4 601 620 10.1089/106652700750050961 11108481

[B32] Feng S Agoulnik IU Bogatcheva NV Kamat AA Kwabi-Addo B Li R Ayala G Ittmann MM Agoulnik AI Relaxin promotes prostate cancer progression Clin Cancer Res 2007 13 6 1695 1702 10.1158/1078-0432.CCR-06-2492 17363522

[B33] Hata S Hamada J Maeda K Murai T Tada M Furukawa H Tsutsumida A Saito A Yamamoto Y Moriuchi T PAX4 has the potential to function as a tumor suppressor in human melanoma Int J Oncol 2008 33 5 1065 1071 18949370

[B34] Robson EJ He SJ Eccles MR A PANorama of PAX genes in cancer and development Nat Rev Cancer 2006 6 1 52 62 10.1038/nrc1778 16397527

[B35] Shih WJ Collins J Mitchell B Wierzbinski B Serum PSA and PAP measurements discriminating patients with prostate carcinoma from patients with nodular hyperplasia J Natl Med Assoc 1994 86 9 667 670 7525979 PMC2607589

[B36] Leaner VD Chick JF Donninger H Linniola I Mendoza A Khanna C Birrer MJ Inhibition of AP-1 transcriptional activity blocks the migration, invasion, and experimental metastasis of murine osteosarcoma Am J Pathol 2009 174 1 265 275 10.2353/ajpath.2009.071006 19074613 PMC2631339

[B37] Tiniakos DG Mitropoulos D Kyroudi-Voulgari A Soura K Kittas C Expression of c-jun oncogene in hyperplastic and carcinomatous human prostate Urology 2006 67 1 204 208 10.1016/j.urology.2005.07.045 16413376

[B38] Gupta R Sharma S Sommers JA Jin Z Cantor SB Brosh RM Jr Analysis of the DNA substrate specificity of the human BACH1 helicase associated with breast cancer J Biol Chem 2005 280 27 25450 25460 10.1074/jbc.M501995200 15878853

[B39] Ono A Kono K Ikebe D Muto A Sun J Kobayashi M Ueda K Melo JV Igarashi K Tashiro S Nuclear positioning of the BACH2 gene in BCR-ABL positive leukemic cells Genes Chromosomes Cancer 2007 46 1 67 74 10.1002/gcc.20390 17044046

[B40] Antunes AA Leite KR Sousa-Canavez JM Camara-Lopes LH Srougi M The role of prostate specific membrane antigen and pepsinogen C tissue expression as an adjunctive method to prostate cancer diagnosis J Urol 2009 181 2 594 600 10.1016/j.juro.2008.10.007 19084862

[B41] Vanhara P Lincova E Kozubik A Jurdic P Soucek K Smarda J Growth/differentiation factor-15 inhibits differentiation into osteoclasts--a novel factor involved in control of osteoclast differentiation Differentiation 2009 78 4 213 222 10.1016/j.diff.2009.07.008 19695766

[B42] Kawahara T Ishiguro H Hoshino K Teranishi J Miyoshi Y Kubota Y Uemura H Analysis of NSAID-activated gene 1 expression in prostate cancer Urol Int 2010 84 2 198 202 10.1159/000277599 20215826

[B43] Wang X Southard RC Allred CD Talbert DR Wilson ME Kilgore MW MAZ drives tumor-specific expression of PPAR gamma 1 in breast cancer cells Breast Cancer Res Treat 2008 111 1 103 111 10.1007/s10549-007-9765-7 17902047 PMC2673095

[B44] Song J Murakami H Tsutsui H Tang X Matsumura M Itakura K Kanazawa I Sun K Yokoyama KK Genomic organization and expression of a human gene for Myc-associated zinc finger protein (MAZ) J Biol Chem 1998 273 32 20603 20614 10.1074/jbc.273.32.20603 9685418

[B45] Voulgari A Voskou S Tora L Davidson I Sasazuki T Shirasawa S Pintzas A TATA box-binding protein-associated factor 12 is important for RAS-induced transformation properties of colorectal cancer cells Mol Cancer Res 2008 6 6 1071 1083 10.1158/1541-7786.MCR-07-0375 18567809

