# Supplementary material for: RegNetB: Predicting Relevant Regulator-Gene Relationships in Localized Prostate Tumor Samples
Source: BMC Bioinformatics. 2011 Jun 17;12:243. doi: 10.1186/1471-2105-12-243 (PMC3128037; doi:10.1186/1471-2105-12-243)
Supplement: Additional file 1 — Bayesian Dirichlet metric. This document includes more details about the BD scoring metric used in this work as well as an explanation of the Gibbs sampler integrated for the scoring in the presence of hidden variables or missing entries in general. [file 1471-2105-12-243-S1.DOCX]

**Supplemental material**

Here we describe the BDe (Bayesian Dirichlet equivalent) scoring metric used in this work. Also, a description of the integrated Gibbs sampling is provided. Additional theoretical details for these numerical/computational tools can be found in the referenced documents provided in the bibliography.

*Static Bayesian networks*

Bayesian networks can be used to describe causal or apparently casual relationships in data. The algorithm for scoring the likelihood of a Bayesian network given data is based on a widely used and theoretically sound principle of probability theory called Bayes’ Rule. Bayes’ rule in this context is used to evaluate the probability that a model is true given a body of experimental data. Mathematically Bayes’ rule can be expressed as:


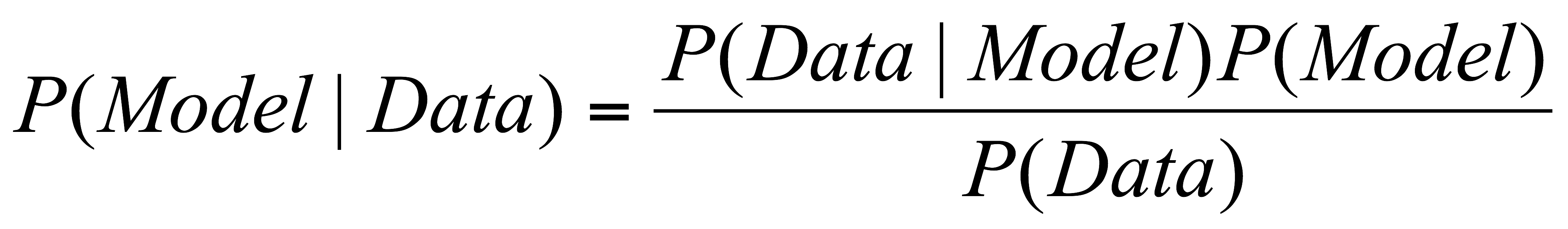


For a Bayesian network, a model is a directed acyclic graph. Nodes in this graph represent variables. Arrows between nodes represent probabilistic dependencies indicating a causal relationship between the two variables. These probabilistic dependencies can be estimated using experimental data or known facts that interrelate the variables. Hence, for each node, there is a conditional probability set of values that quantitatively describes the relationship between the node and its descriptors (parents).

Note that the graphical representation of a Bayesian network is similar to that of a kinetic model such as a signaling pathway, but is interpreted differently. In a kinetic model, edges represent a specific function (activation, repression, a linear relationship, etc.), or a transformation (e.g. A -> B implies A becomes B). In a Bayesian network, these causal relationships may be any activation effect as well as inhibition and also includes linear, nonlinear, and/or multimodal associations between variables.

The term P(Model | Data) represents the probability that the model is correct given the observed data. P(Data) is not calculated as it is a constant in our expression, thus we will only compare relative scores. In the RegNetB analysis, P(Model) was either 1 or 0 for networks that were and were not allowed respectively.

The P(Data | Model) is the probability of the particular data configuration given the model. This term is calculated by marginalizing over all parameters in a specific model (conditional probability values associated with each node). In this work, connections between a gene and its regulator are modeled as a discrete multinomial distribution with Dirichlets priors. By using a multinomial model, the network can capture both linear and non-linear relationships. In addition, for a multinomial model, the term P(Data | Model) has a closed form solution described elsewhere [8, 10]. This solution is known as the Bayesian Dirichlet Equivalent metric (BDe) and has the following form:


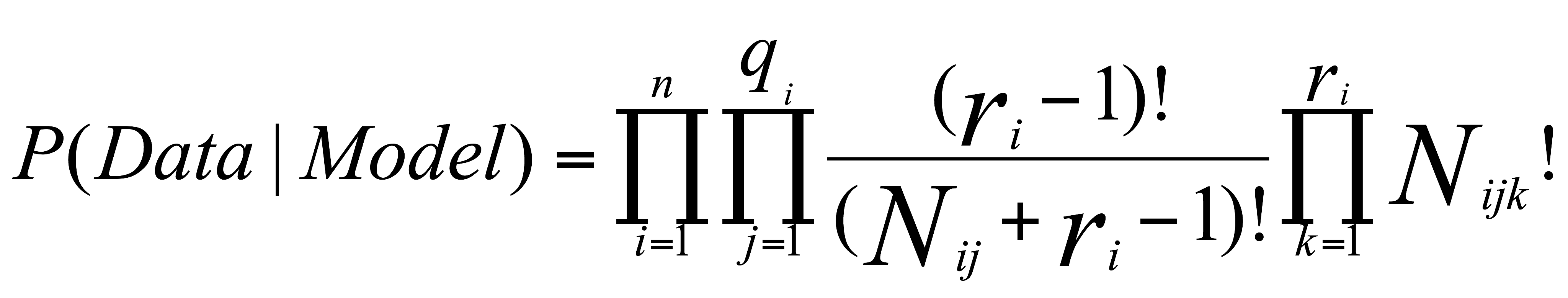
 (1)

Where “*n”* is the total number of variables, “*qi*” is the total possible state configurations for a parent, “*ri*” is the number of states of a variable (arity), “*Nij*” is the number of cases parent of variable “*i”* is in state (or state combination) “*j*”, “*Nijk*” is the number of cases variable “*i*” is in state “*k*” and parent(s) in state “*j*”. The expression in equation (1) describes the product of the probability of a variable being in a state *k* and the parents of this variable in a state *j*. The more informative the parents are of their child, the higher the value of P(Data | Model).

Because we are modeling our regulatory models as a bipartite network, network learning and scoring for RegNetB is simpler than the general Bayesian Networks learning problem. Below we describe how RegNetB handles the missing values for the regulatory proteins.

*Estimating a BDe metric value (network score) with missing data*

A key challenge in identifying regulatory relationships is the lack of data on the activity of the regulators themselves. With these values missing, we used a modified method to estimate the score of a network where the activity of the regulator is assumed to be unknown. A simple but computationally unfeasible way to evaluate the score of a network with missing values is to marginalize over all possible state configurations for the missing data and then take an average. However, the number of possible state configurations increases exponentially with the number of missing values, making this exact marginalization impractical. For example, in a small system with 2 missing binary variables and 10 observations there are more than a million possible different state configurations.

An alternative to exact enumeration is to selectively sample the configuration space. To do this sampling, we used an MCMC method known as Gibbs sampling. Gibbs sampling is commonly used in computational statistics, and has found extensive use in missing value estimation on Bayesian networks [21-24]. In general, Gibbs sampling works in the following way:

• Values for all unobserved entries are randomly chosen each time that a BDe metric value needs to be estimated

• A randomly chosen unobserved entry is re-sampled based on the probability of each of the states for the visited variable as calculated with P(Model | Data).

• The new sampled value for an entry is used when evaluating a future entry.

The last two steps are repeated many times. Note that Gibbs sampler does not select a single best data configuration, but instead samples a wide variety of possible configurations for the hidden values, favoring the more likely configurations over the less likely ones. Also, in our case, the method only sampled missing data configurations that result in a maximum entropy discretization for the hidden variables as this was the discretization scheme used for all the variables in the data set.

The result of this calculation is an average probability score of the model given the available data.
